# COVID-19 mortality in Lombardy: the vulnerability of the oldest old and the resilience of male centenarians

**DOI:** 10.18632/aging.103872

**Published:** 2020-08-12

**Authors:** Gabriella Marcon, Mauro Tettamanti, Giorgia Capacci, Giulia Fontanel, Marco Spanò, Alessandro Nobili, Gianluigi Forloni, Claudio Franceschi

**Affiliations:** 1 DAME, University of Udine, Udine, Italy; 2 Azienda Sanitaria Universitaria Giuliano-Isontina (ASUGI), Trieste, Italy; 3 Department of Medical Surgical and Health Sciences, University of Trieste, Trieste, Italy; 4 Department of Neuroscience, Istituto di Ricerche Farmacologiche Mario Negri IRCCS, Milan, Italy; 5 Directorate for Social Statistics and Population Census, Istat, Italy; 6 Department of Experimental, Diagnostic and Specialty Medicine (DIMES), University of Bologna, Bologna, Italy; 7 Laboratory of Systems Medicine of Healthy Aging, Lobachevsky State University of Nizhny Novgorod, Nizhny Novgorod, Russia

**Keywords:** centenarians, inflammaging, COVID-19, mortality, oldest-old

## Abstract

Italy was the first European nation to be affected by COVID-19. The biggest cluster of cases occurred in Lombardy, the most populous Italian region, and elderly men were the population hit in the hardest way. Besides its high infectivity, COVID-19 causes a severe cytokine storm and old people, especially those with comorbidities, appear to be the most vulnerable, presumably in connection to inflammaging. In centenarians inflammaging is much lower than predicted by their chronological age and females, presenting survival advantage in almost all centenarian populations, outnumber males, a phenomenon particularly evident in Northern Italy. Within this scenario, we wondered if: a) the COVID-19 mortality in centenarians was lower than that in people aged between 50 and 80 and b) the mortality from COVID-19 in nonagenarians and centenarians highlighted gender differences.

We checked COVID-19-related vulnerability/mortality at the peak of infection (March 2020), using data on total deaths (i.e. not only confirmed COVID-19 cases). Our conclusion is that excess mortality increases steadily up to very old ages and at the same time men older than 90 years become relatively more resilient than age-matched females.

## INTRODUCTION

Italy was the first European country to suffer from the COVID-19 pandemic. A main characteristic of this pandemic, besides high infectivity of its causative agent, is a cytokine storm characterized by an IL-6 centered response [1, 2] and the uneven distribution of severity and mortality among different age classes. Indeed, old people, and particularly those with one or more comorbidity, appear to be the most vulnerable [3, 4]. The reasons of such a high vulnerability to COVID-19 is poorly understood but it has been suggested that a major role is played by inflammaging [5–7], i.e. the low-grade chronic inflammation that is a major driver of aging [8] and whose basic underlying mechanisms are shared with those responsible for frailty and age-related diseases (ARDs), including cardiovascular disease, type 2 diabetes, chronic obstructive pulmonary disease, chronic kidney disease and dementia, among others [9–11]. However, a major characteristic of old people is their heterogeneity regarding not only their health status (presence/absence of comorbidities, frailty, cognitive status) but also, in particular, their different capability to mount an immune response to pathogens and vaccines [12, 13]. At present it is possible to quantify such heterogeneity using a variety of proteomic [15] and epigenetic biomarkers [16] capable of distinguishing between chronological and biological age, and to predict the risk of developing major ARDs. In particular, we showed that centenarians, i.e. subjects who avoided or largely postponed all major ARDs, and their offspring, are characterized by being healthier than age-matched controls born from non-long-living parents, i.e. a slower aging and are biologically younger than their chronological age of about 9 and 5 years, respectively. Particularly important within the scenario of COVID-19 pandemic is that centenarians have a peculiar state/degree of inflammaging, which is much lower than that predicted by their chronological age and is biased toward anti-inflammaging, i.e. the production of anti-inflammatory molecules and cells that the body produces lifelong as an adaptive, compensatory mechanisms to continuously down-regulate the inflammatory process and avoid its chronic detrimental effects [18–20]. Accordingly, the oldest old, including centenarians, are high-selected, exceptionally robust subjects that can be taken as a model of successful/healthy aging [21].

A major characteristic of human longevity is the ubiquitous female survival advantage. In particular, centenarian females outnumber males [22], and this demographic phenomenon is particularly evident in Northern Italy, including Lombardy [23]. However, although women live longer, they suffer greater morbidity, particularly late in life. In Trieste, a city situated in the North-East of Italy with 204,000 inhabitants, the prevalence of centenarians is high: in mid-June 2020 there were 148 centenarians, number obtained from the list of the public health service considering only subjects who have reached 100 years of age. In 2014 we started the Centenari a Trieste (CaT) Study, to examine the centenarians living in Trieste. From 2014 to January 2020 we enrolled, visited and collected data of 130 centenarians, using the annual lists provided by the public health service mentioned above. 90% of our centenarian population are women, but the few males are all in excellent health [24]. The complex reasons of such a female longevity advantage/paradox is still unclear [25, 26] but it is likely the result of a mixture of biological (e.g. genetics) and non-biological (e.g. cultural, anthropological) factors [27, 28].

Within this scenario, and considering that the population age 100 years and older is part of the fastest growing segment of the population worldwide, we thought worthwhile to check COVID-19-related vulnerability/mortality in old people across the above-mentioned large and heterogeneous age spectrum, focusing on nonagenarians and centenarians and gender, in Lombardy, the largest (10 million inhabitants) and most populous Italian Region, heavily affected by COVID-19 pandemic. To this regard, our study refers to the peak of infection (March 2020) when the number of (reported) infected people was 76,586 and the number of deaths was 11,399 (data from the Italian Civil Protection Department available at: http://opendatadpc.maps.arcgis.com/apps/opsdashboard/index.html-/b0c68bce2cce478eaac82fe38d4138b1).

The following questions/ hypotheses were addressed/ tested: i) is the COVID-19-related mortality of exceptionally long-living subjects, lower than that of people in the age-range between 50 and 80 years of age? ii) do the COVID-19-related mortality data show any gender difference in nonagenarians and centenarians?

## RESULTS

### Lombardy municipalities

During March 2020 a large increase in mortality was seen in Lombardy relative to previous years, both in absolute and in relative terms: against a background of 8492 deaths (mean of March deaths between 2015 and 2019), in 2020 there were 24,330 deaths, constituting an increase of 15,838 in absolute numbers and of 286% in percentage. Men contributed more to this increase with 9021 (57.0%) extra deaths. Increase in mortality is apparent in older age groups. In fact, while excess mortality under 40 years of age totalized less than 50 persons, its maximum was reached in the 80-84 and 85-89 age categories, with about 3300 more deaths each (Supplementary Figure 1).

**Figure 1:**
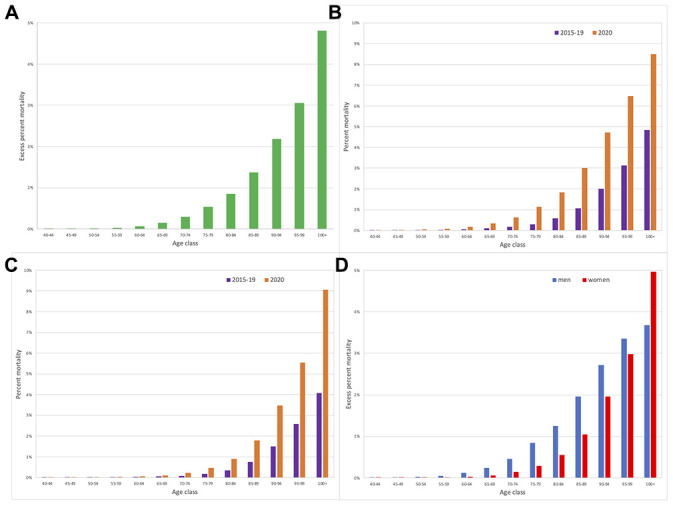
**Mortality in March 2020 in Lombardy compared with mean mortality in March in 2015-2019.** (**A**) Percent March 2020 excess mortality, by age class. (**B**) Men percent mortality by age class and year. (**C**) Women percent mortality by age class and year. (**D**) Percent March 2020 excess mortality by age class and sex.

When percent excess death by age class was plotted, a continuous increase in mortality by age was apparent (Figure 1, panel A). This phenomenon was clearly visible in both men (Figure 1, panel B) and in women (Figure 1, panel C), where March 2020 mortality is compared with mean March mortality of the previous years. However, the two patterns had also some differences: in women the increase resulted in approximately a doubling in mortality risk in each age class, whereas in men the greatest relative increase was in "younger" ages, were 2020 mortality was more than three times that of previous years, while in later ages the increase is about 80%. These different changes result in two different patterns of increase by age, i.e. women increase in excess mortality was lower in "younger" ages, but reached that of men in later ages (Figure 1, panel D): while excess mortality under 90 was much higher in men, it was similar in the nonagenarians and in centenarians women even had a higher mortality.

We tested if the increase in mortality by age was different between men and women entering an interaction (age*sex) term in a logistic model: the effect was statistically significant (p<0.0001). We further refined the model entering age also as a quadratic term, together with its interaction with sex (age squared*sex): the interaction term resulted statistically significant (p<0.0001). This latter model was statistically better than the simpler one (p<0.0001). Both models indicated that the probability of dying was much higher in "young" men, but that at older ages the difference was less pronounced (simpler model) or even reversed (model with quadratic age).

### Mortality in Trieste

Owing to the above-mentioned large heterogeneity of the health status of elderly subjects including nonagenarians and centenarians [12–14], and considering that morbidity, mortality and longevity outcomes are largely context-dependent [27, 28], it is interesting to look at what can be observed with a higher “granularity”.

**Figure 2:**
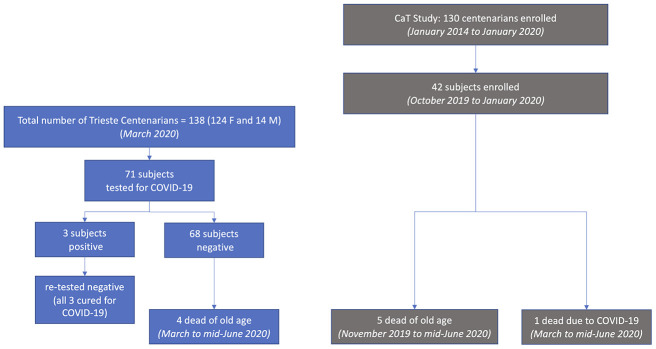
**COVID-19 testing and deaths in Trieste (left) and in the CaT (Centenari a Trieste) Study (right).**

In March 2020 in Trieste there were 138 centenarians, 90% of them were women (Figure 2). 71 centenarians were tested with swab for COVID-19: three of them resulted positive but subsequently became negative at test and were therefore considered cured of COVID-19 infection. The remaining 68 centenarians tested negative for COVID-19: four of them died of old age from March to mid-June 2020.

As part of the CaT Study, from October 2019 to January 2020, immediately before medical emergency for COVID-19, we enrolled 42 centenarians using the list of the public health service, 39 women and 3 men (Figure 2). Six centenarians died before April 2020: five women for senectus and without symptoms related to COVID-19 infection. A man was admitted in a ward COVID-19 infected and was the only one dying with COVID-19 pathology. A woman of the 42 centenarians enrolled in the Study tested for COVID-19 was negative despite living in a nursing home with a COVID-19 outbreak, where most of the other elderly guests became positive. She was one of the centenarians belonging to the group of 68 tested negative and mentioned above.

## DISCUSSION

The answer to the first question (is the COVID-19-related mortality of exceptionally long-living subjects lower than that of people in the age-range between 50 and 80 years of age?) is negative. In a region such as Lombardy which experienced a high SARS-CoV-2 infection rate, we found a continuous increase in mortality by age when percent excess death by age class was plotted. On the whole, nonagenarians and centenarians, despite their capability to survive until an extreme age and to avoid/postpone most of the ARDs, could be highly vulnerable during personal and societal stressful events like as seen during the SARS-CoV-2 pandemic. These data are in accord with the hypothesis that a major reason of such increasing vulnerability of the elderly, including nonagenarians and centenarians, to COVID-19 infection and related stressful conditions is inflammaging, the age-related increase of the inflammatory status which is particularly deleterious in those old subjects affected by one or more comorbidities. Inflammaging is a complex phenomenon at present only partially understood which can be highly different and personalized in different individuals [29]. Accordingly, the conceptual framework of inflammaging could help in understanding both the higher vulnerability of the elderly to COVID-19 but also the different responsiveness to COVID-19 infection and related contextual stressors in different subsets of elderly people.

The take home message is that nonagenarians and centenarians need particular attention, protection and special care in situations challenging the capability of hospitals, nursing homes and Health Service to cope with exceptional events like the COVID-19 pandemic.

However, when mortality is disentangled according to gender a peculiar gender-specific crossing emerged. The excess of mortality presumably due, directly or indirectly, to COVID-19 explosively grew in males from 50 years of age up to 80 years but thereafter the rise tended to slow down. Females had a similar age trend, but their risk was lower in lower ages than in males and the decrease in higher age groups was less marked. Thus, very old people such as nonagenarian and centenarian males appears to be more resilient than age-matched females.

Accordingly, the answer to the second question (do the COVID-19-related mortality data show any gender difference in nonagenarians and centenarians?) is positive.

The reasons of such gender-specific trajectories of resilience are unclear. We reported that in men a genetic predisposition to produce high levels of IL-6 is detrimental for longevity [30]. In subjects with ages ranging from 22 to 93 years the age-related decline in adaptive immunity (particularly T cells) and especially the activation of innate immunity despite being present in both women and men were significantly greater in magnitude in men, suggesting that they experience a stronger inflammaging than women even when the subjects were otherwise healthy and clinically comparable in terms of age, BMI, and ethnicity [31]. Men have also a stronger inflammatory state in circulating monocytes compared to women [31]. Thus, men-specific immune characteristics interacting with/related to inflammaging, such as a blunted acquired immune system and type I interferon response, coupled with the downregulation of ACE2 (SARS-CoV-2 receptor) (particularly in patients with age-related comorbid diseases such as type II diabetes) and an accelerated biological aging (measured by epigenetic markers and telomere shortening), could help in explaining the higher vulnerability of men to COVID-19 infection [32].

To understand why men older than about 90 years become relatively more resilient than age-matched females it is important to consider the above-mentioned female-male health-survival paradox [25, 26, 33]. Indeed, despite women live longer than men and appear to be stronger even during severe famines and epidemics [34] when they became nonagenarians and centenarians show a much worse health status than that of nonagenarian and centenarian men who have a much better physical and cognitive health. The more years of life expectancy of women are mostly years of disease and disability [27, 28]. In any case, nonagenarians and centenarians are a mix of those aging well and those aging poorly, and in this heterogeneous scenario men capable of reaching age 90 and especially 100 are likely the more robust. Centenarian men are fewer but more selected and healthier and likely more resilient than centenarian women in highly stressful conditions like COVID-19 pandemic.

Finally, it is important to note that the two general conclusions of our study, i.e. the high COVID-19-related mortality of nonagenarians and centenarians and the relative resilience of male centenarians, resulting from epidemiological investigations can be at variance with anecdotal observations that centenarians and sometime supercentenarians (people over 110 years old) survived and recovered after COVID-19 infection. As an example, our data on a low number of very well characterized subjects of the CaT Study, suggest that both centenarian women and men looked strong during the peak of COVID-19 pandemic which profoundly challenged the entire health system and care of the elderly. What can be observed and reported at a higher magnification and higher granularity in single cities, institutions and settings is the consequence of the basic heterogeneity of the aging phenotype which is particularly evident at the extreme ages and suggests that outcomes may differ by robustness or other characteristics of the individual and are always highly diverse and context-dependent. To this regard, it can be predicted that the use of proteomic [15], epigenetic [16, 17] and glycomic biomarkers [35], among others, capable of distinguishing between chronological and biological age, will help in disentangling the heterogeneity of the aging phenotypes and in identifying the elderly characterized by an accelerated aging and lower robustness and thus at higher risk of morbidity and mortality in normal as well in exceptional circumstances such the COVID-19 pandemic.

### Strength and limitations

We had access to open data provided by Istat, which despite being a non-representative subset of Italian municipalities covers the Lombardy population almost completely (about 97%). The data on the entire Italian population would have diluted the results here presented, owing to the much lower mortality in the other Italian regions.

We analyzed total mortality and not COVID-19-related deaths. This is both a limitation and a strength. Due to the great strain imposed on the Italian National Health Service, particularly in the hardest hit provinces, we cannot exclude an increase in general mortality due to a missing response to needs that would have been otherwise met. Even if this may not be excluded, we find difficult to think of logistical reasons that would differentially impact men and women and spare oldest men. Analyzing only confirmed COVID-19 deaths is more specific but, due to the impressive surge in mortality, only a part of those who died due to the infection were reported as being infected, and only a part of them was subject to a verification. Also, in absence of clear typical manifestations, a part of COVID-19 mortality could be incorrectly attributed to other causes, even after a closer reanalysis, since swabs were only partially available, and their sensitivity is far from perfect.

In conclusion, we reported data that clearly show that old people, including nonagenarians and centenarians, suffered a high COVID-19-related mortality in the Lombardy region and suggested that the conceptual framework of inflammaging could help in understanding such age-related vulnerability. The remarkable difference between women and men in life expectancy, disability, mortality and longevity which emerged also in circumstances such as the COVID-19 pandemic is complex but still poorly understood and deserves attention and a closer scrutiny. Preventive strategies focused on the elderly preparing us better for the next pandemic are urgently needed [6].

## MATERIALS AND METHODS

We used publicly available online data from the Istat (Italian Institute of Statistics) site: https://www.istat.it/it/archivio/240401 (accessed on June 15, 2020). Mortality raw data in a large dataset of Italian municipalities were collected by ANPR (National Registry of Resident Population) operated by the Ministry of the Interior. These data were successively merged with the dataset of the Registry Tax operated by the Ministry of Economy and Finance, validated and made available on-line by Istat.

Mortality data were made available for each day starting from January 1 to Apr 30, 2020 by municipality, 5-year age classes, and sex. Reference mortality data are available for the years 2015 to 2019, with same granularity.

Since we wanted to study the effect of the virus on mortality by age we concentrated on the Lombardy region, which presently (June 15, 2020) accounts for almost half of the confirmed COVID-19 deaths in Italy, and on the peak of infection (March 2020). Notably the dataset covers 97.1% of the Lombardy population.

Population in Italy (and as a consequence also in Lombardy) is gradually ageing, rendering impossible a direct comparison of 2020 deaths to 2015-2019 deaths. In order to correct for this imbalance we calculated the 2020 death percentage comparing the number of deaths within age classes with the respective age class populations, i.e. [(March 2020 number of deaths)/(March 2020 Lombardy population)]*100, for each age class, by sex. Reference 2015-2019 death percentage was calculated similarly as [(mean March 2015-2019 number of deaths)/(mean March 2015-2019 Lombardy population)] *100, for each age class, by sex. Percent excess mortality was calculated as a difference between 2020 mortality percentage and previous years mean mortality percentage. Lombardy population data was retrieved from the demo.istat.it site. Population data for 2020 is not available yet, so we used the data from the Istat population projections for 2020 available from same site.

We used logistic regression models in which age and sex were used as predictors for March 2020 probability of excess mortality, i.e. we disregarded "usual" (mean 2015-2019) number of March deaths. Age was modelled as a continuous factor, and age classes were given an intermediate value: for example 80-84 class was given an 82.5 value. Last class (100+) was given a 102.5 value. Models tried were hierarchically related: first only age and sex, then an interaction age*sex was entered into the model to test if the rate of increase was different between sexes. A quadratic effect, and its interaction was tried in a subsequent model.

The protocol of the CaT study to obtain, after informed consent, demographic, anamnestic, clinical and lifestyle data from the subjects enrolled in the study was already published [36]*.*

JMP Pro v 15.0 (SAS Institute Inc.) was used to manage data and perform statistics.

## Supplementary Material

Supplementary Figure 1
