# Supplementary material for: COVID-19 mortality in Lombardy: the vulnerability of the oldest old and the resilience of male centenarians
Source: Aging (Albany NY). 2020 Aug 12;12(15):15186–95. doi: 10.18632/aging.103872 (PMC7467374; doi:10.18632/aging.103872)
Supplement: Supplementary Figure 1 [file aging-12-103872-s001..pdf]

SUPPLEMENTARY FIGURE

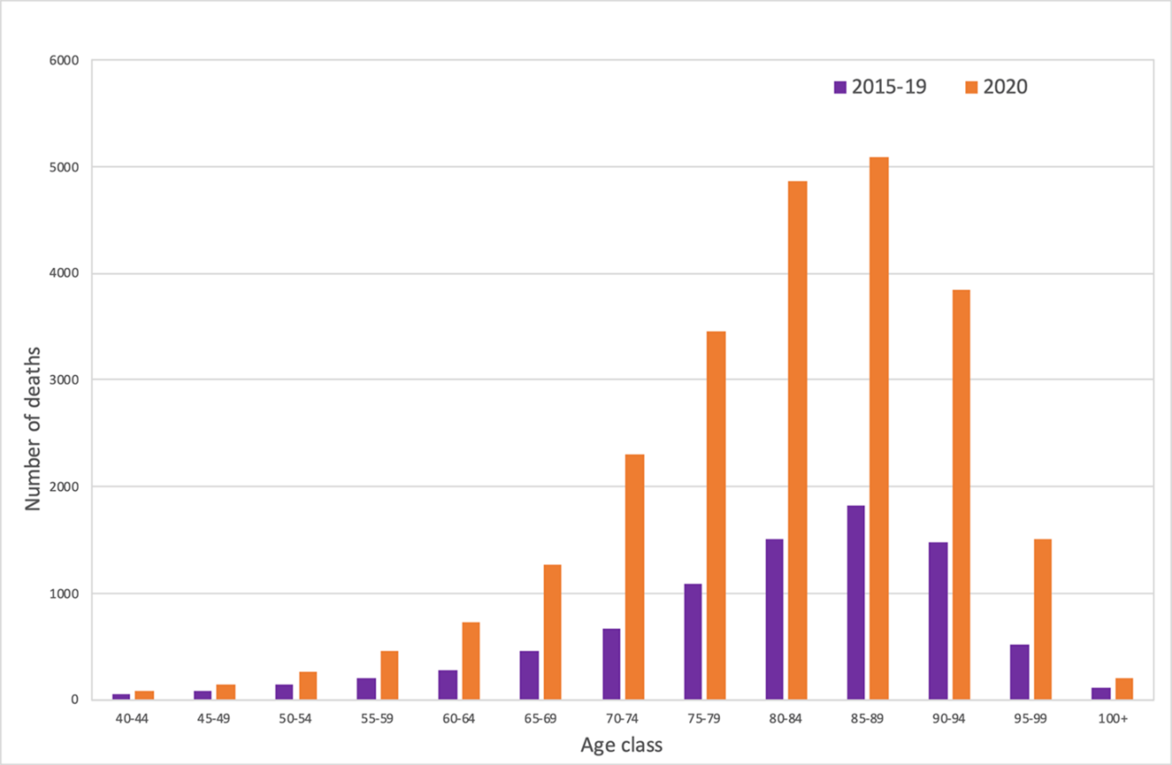

Supplementary Figure 1. Total number of deaths in March in Lombardy, by age class and year.
